# Ligament, hinge, and shell cross-sections of the Atlantic surfclam (*Spisula solidissima*): Promising marine environmental archives in NE North America

**DOI:** 10.1371/journal.pone.0199212

**Published:** 2018-06-14

**Authors:** Pierre Poitevin, Julien Thébault, Bernd R. Schöne, Aurélie Jolivet, Pascal Lazure, Laurent Chauvaud

**Affiliations:** 1 Université de Bretagne Occidentale, Laboratoire des Sciences de l'Environnement Marin (UMR6539 UBO/CNRS/IRD/Ifremer), Plouzané, France; 2 Institute of Geosciences, University of Mainz, Johann-Joachim-Becher-Weg 21, Mainz, Germany; 3 TBM environnement/Somme, Technopole Brest-Iroise, Plouzané, France; 4 Ifremer, Laboratoire d'Océanographie Physique et Spatiale (UMR6523 CNRS/Ifremer/IRD/UBO), Plouzané, France; University of California, UNITED STATES

## Abstract

The Atlantic surfclam (*Spisula solidissima*) is a commercially important species in North American waters, undergoing biological and ecological shifts. These are attributed, in part, to environmental modifications in its habitat and driven by climate change. Investigation of shell growth patterns, trace elements, and isotopic compositions require an examination of growth lines and increments preserved in biogenic carbonates. However, growth pattern analysis of *S*. *solidissima* is challenging due to multiple disturbance lines caused by environmental stress, erosion in umbonal shell regions, and constraints related to sample size and preparation techniques. The present study proposes an alternative method for describing chronology. First, we analyzed growth patterns using growth lines within the shell and hinge. To validate the assumption of annual periodicity of growth line formation, we analyzed the oxygen isotope composition of the outer shell layer of two specimens (46°54'20"N; 56°18'58"W). Maximum δ^18^O_shell_ values occurred at the exact same location as internal growth lines in both specimens, confirming that they are formed annually and that growth ceases during winter. Next, we used growth increment width data to build a standardized growth index (SGI) time-series (25-year chronology) for each of the three parts of the shell. Highly significant correlations were found between the three SGI chronologies (*p* < 0.001; 0.55 < τ < 0.68) of all specimens. Thus, ligament growth lines provide a new method of determining ontogenetic age and growth rate in *S*. *solidissima*. In a biogeographic approach, the shell growth performance of *S*. *solidissima* in Saint-Pierre and Miquelon was compared to those in other populations along its distribution range in order to place this population in a temporal and regional context.

## Introduction

The Atlantic surfclam (*Spisula solidissima*) is the largest bivalve in the western North Atlantic, reaching a maximum length of 226 mm (commercial minimum size: 120 mm in USA and 90 mm in Canada) and longevity of 37 years in the Middle Atlantic Bight population [1]. *S*. *solidissima* is a commercially important species in Canada and the US Exclusive Economic Zone (EEZ). The US fishery represents nearly 75% of Atlantic surfclam global landings between 1965 and 2011. In 2011, approximately 20 000 tons of Atlantic surfclam meats were landed, 93% of which came from the US EEZ, corresponding to nominal revenues of $29 million, making this fishery one of the most valuable single species fisheries in the US [2].

*S*. *solidissima* is a good example of a commercially important species undergoing biological and ecological changes that have been attributed to increased bottom water temperature, the fishery activity, or a combination of both [3–8]. These data are measured within the accretionary hard parts of the clam [9–11]. Shell growth, a variable that integrates multiple physical and biological factors, represents an integrative approach to monitoring the impact of environmental changes in *S*. *solidissima* populations along a geographic gradient during the last few decades [7, 12–14].

Previous studies have reported that *S*. *solidissima* is an aragonitic bivalve [15] that forms one growth line per year during fall [9]. Based on this observation, different methods have been used to measure growth rates in Atlantic surfclam shells, including the size distribution of single cohorts [16], analysis of growth increments following mark-and-recapture experiments using different labeling techniques [17], external shell growth line measurements [18], internal growth line analysis in shell cross-sections [1], and elemental and stable oxygen isotope analyses [19–21]. However, disturbance rings caused by storms, thermal stress, predators, diseases, spawning, gonad development, and dredging are often indistinguishable from (periodic) annual growth lines, leading to unreliable results [9, 22]. Further limitations occur in older specimens, in which it is sometimes a bit more difficult to resolve the most recently formed growth lines and the umbonal region may be eroded. In addition, the cutting, polishing, and examination procedures are considered to be time-consuming [19]. In order to resolve some of these problems, [19] proposed another method for determining the age and growth rate using internal growth lines preserved in the chondrophore, a structure that is particularly well developed in members of the Mactridae family. This method, which was improved by John W. Ropes [23, 24], is still used every 2–3 years on surfclams sampled in the framework of the NEFSC clam surveys [2]. Although this method has solved the problems related to outer shell layer degradation and the time required, the problems related to disturbance lines persist [24].

**Fig 1:**
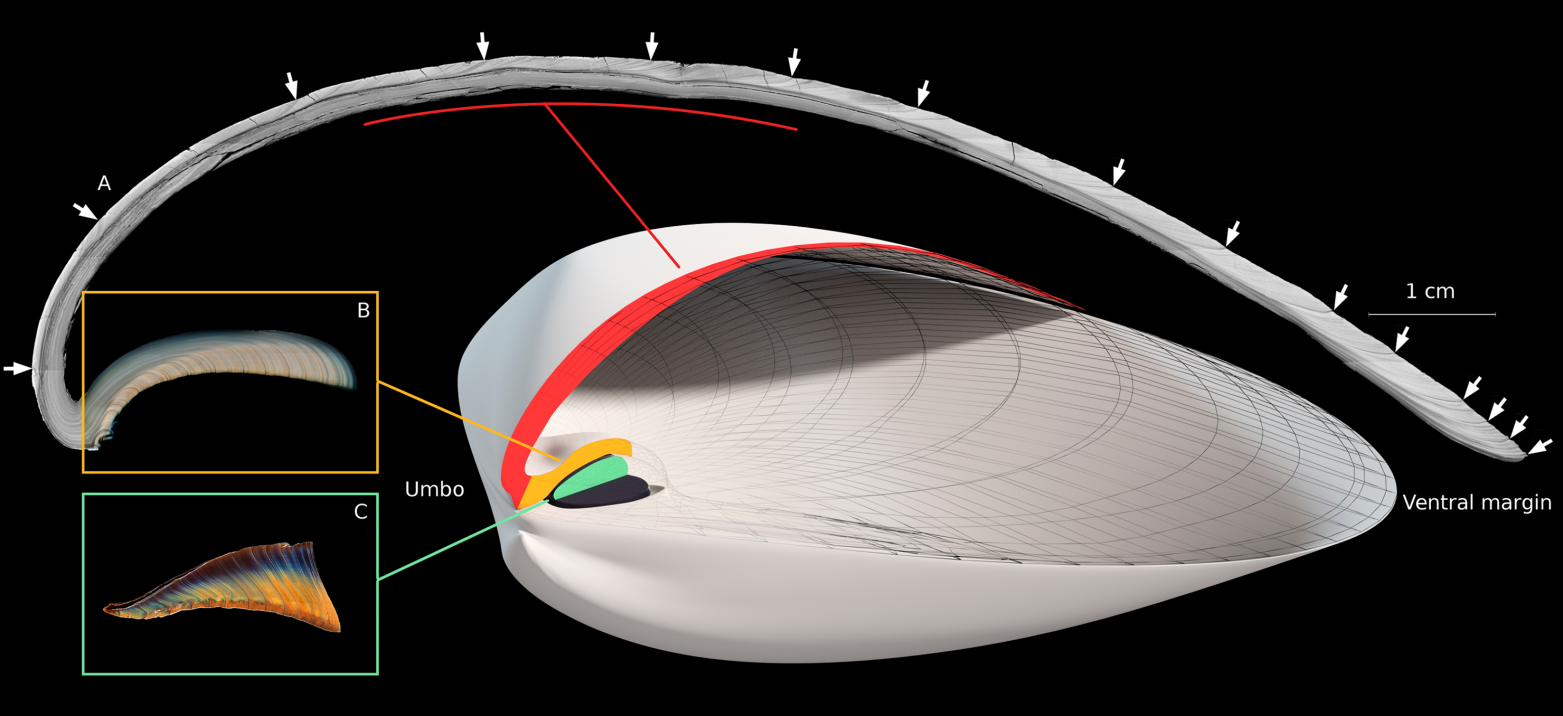
3D model of the general structure of the shell and hinge ligament of *S*. *solidissima*. The outer shell layer (A—red) and chondrophore (B—yellow) of the right valve, as well as the right half of the hinge ligament (C—green), are sectioned along the axis of maximal growth (from the umbo to the ventral margin). White arrows are placed at the locations of annual lines in the outer shell layer. These three structures are associated with the corresponding photomosaic, all of which were obtained from the same individual.

The present study analyzed growth lines present in the outer layer of the shell and the chondrophore and compared them to those readily observed in the internal ligament (resilium) of Atlantic surfclam shells. A strong relationship has been identified between growth patterns in the shell and ligament in several bivalve species, including *Placopecten magellanicus*, *Pedum spondyloideum*, *Radiolites angeoides*, and *Crassostrea gigas* [25–29]. *S*. *solidissima* has two physically separated ligaments: a small external uncalcified ligament (tensilium) and a larger internal partially calcified ligament (resilium) attached to the chondrophore [30]. In the rest of this article the hinge ligament refers to the elastic part composed of oriented aragonite crystals in a protein matrix that connects the shell valves dorsally (resilium). Our study focused on *S*. *solidissima* from Saint-Pierre and Miquelon (SPM), one of the northernmost populations along the eastern coast of North America. Despite the relevance of investigating the growth dynamics of a given species close to the limits of its ecological distribution, to the best of our knowledge, no investigation has yet been conducted on *S*. *solidissima* in SPM. Moreover, the SPM archipelago is free of any commercial and recreational surfclam exploitation. Thus, the objectives of this study were to gain insights into the seasonal dynamics of *S*. *solidissima* shell growth cessation in SPM using oxygen isotope thermometry in the outer shell layer, compare the growth lines present in the outer layer of the shell, the chondrophore, and the resilium (Fig 1), and give SPM population growth results a temporal and regional perspective.

## Materials and methods

### Sampling

**Fig 2:**
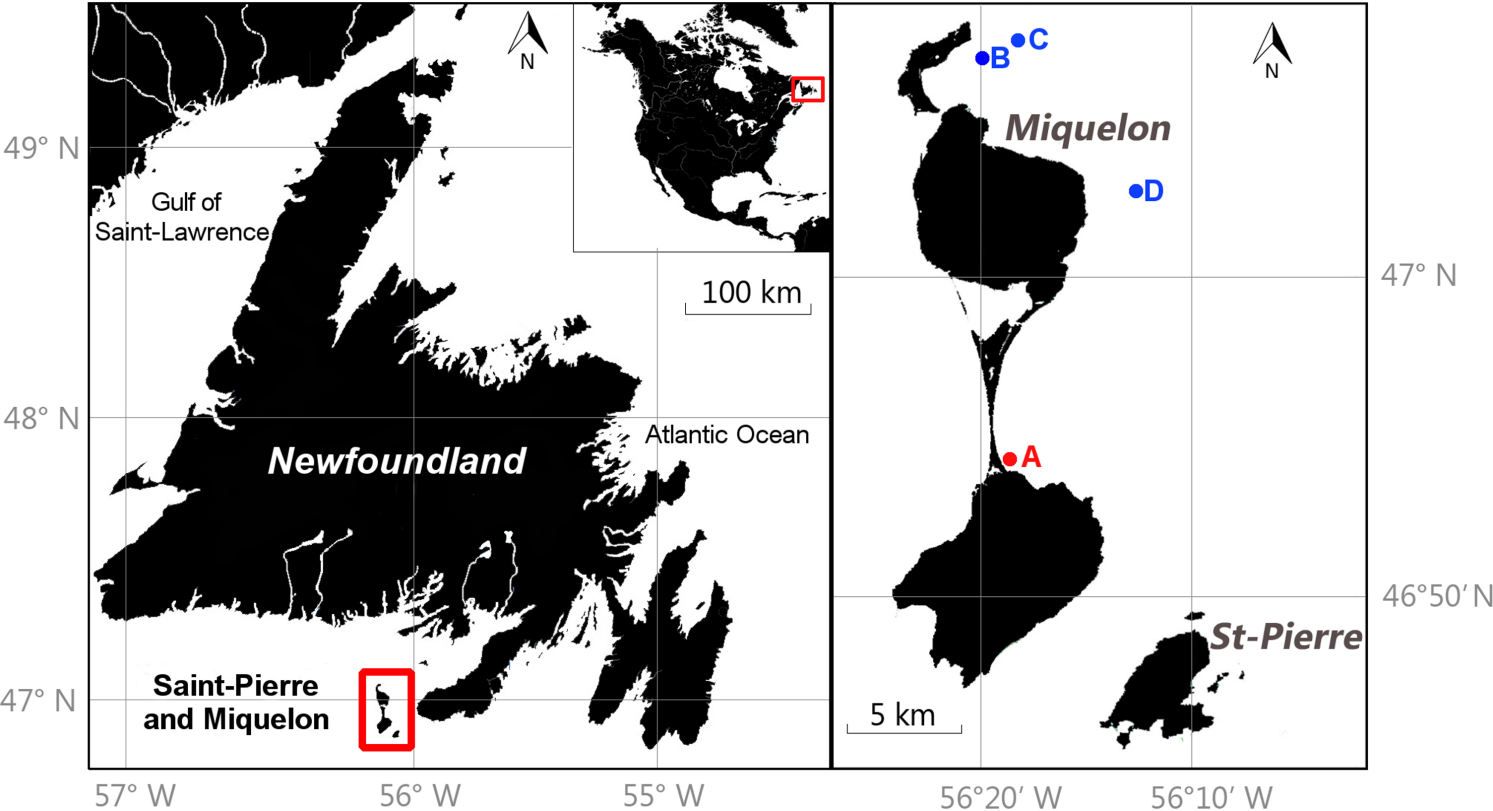
Map of Saint-Pierre and Miquelon and neighboring countries. (A) Location of the *S*. *solidissima* sampling site. (B and C) The two temperature and pressure monitoring sites. (D) The vertical salinity profile site.

Twenty-seven *S*. *solidissima* specimens (shell length ranging from 130 to 171 mm) were analyzed in the present study. They were collected alive at a depth of 0–5 m by scuba diving along the southeastern shore of Miquelon-Langlade sandy isthmus (46°54'20"N; 56°18'58"W) in November 2015 (site A on Fig 2). The habitat at the sampling station consisted of compacted and stable fine sand. Soft tissues, except the hinge ligament, were removed from the live-collected shells immediately after collection. All specimens were carefully cleaned with freshwater to remove adherent sediment and biological tissues.

### Environmental monitoring

Two multi-parameter probes measuring temperature and pressure every 15 minutes were deployed at a depth of 5 m on two ropes moored at a water depth of 30 m and 60 m, respectively, at Miquelon Bay outlet (sites B and C on Fig 2, respectively). A daily average temperature was calculated for the periods 12/07/2010 (day/month/year)-29/11/2010 and 15/06/2011-03/11/2011. Vertical profiles of salinity from a CTD cast run at site D (Fig 2) were performed at water depth of 0–40 m on 04/08/2010, 15/09/2010, 04/10/2010, and 16/11/2010. Measurements were taken at 10 s intervals. We assessed the mean salinity at this time of the year between 0 and 5 m.

### Sample preparation and image analysis

After removal of the ligament, the right valves were embedded in a thin layer of metal epoxy resin (Araldite Metal, Huntsman Advanced Materials) along the axis of maximal growth, from the umbo to the ventral margin. These reinforced parts of the shells were cut using a robust tile saw. Thick cross-sections were then embedded in a polyester mounting resin (SODY 33, ESCIL) to prevent cracking during sectioning. A thin cross-section (2 mm thickness) was cut along the axis of maximum growth using a low-speed precision saw (Struers, Secotom 10; rotation speed 500 rpm; feed rate 200 μm s^−1^) equipped with a 600-μm-thick diamond-coated blade continuously cooled by deionized water. Thin sections were carefully ground on a rotating polishing table (Struers, TegraPol-35) with a sequence of 800, 1200, 2500, and 4000 grit wet-table carborundum paper, followed by polishing with 3-μm diamond liquid (Struers) to remove any saw marks. Cross-sections were ultrasonically cleaned with deionized water between each grinding or polishing step to remove residual abrasive material.

To study the ligament growth lines, the right half of dry hinge ligaments were embedded in a polyester mounting resin (SODY 33, ESCIL). A 2-mm cross-section was cut along the axis of maximum growth using a low-speed precision saw (Struers-Isotom 50; rotation speed 500 rpm; feed rate 100 μm s^−1^) equipped with a 400-μm-thick diamond-coated blade continuously cooled by deionized water. No additional treatment was performed on these sections.

Shell and ligament sections were imaged under reflected light (Zeiss, KL 2500 LCD) using an AxioCam MRc5 installed on a Zeiss Lumar.V12 stereomicroscope equipped with a motorized stage (Fig 1). The outer shell layer sections were photographed under 10x magnification, chondrophores and ligaments under 25x magnification. Photomosaics were constructed using AxioVision 4.9.1 software (Zeiss). The width of each growth increment was measured digitally using the image processing and analysis software Image J (NIH Image).

### Isotopic validation of annual banding

Seawater oxygen isotope composition is controlled by the balance between evaporation and precipitation. Due to their offshore island status, SPM is not subject to major riverine inputs and associated variations in salinity. Moreover, the calcium carbonate phase of most bivalve mollusks is in oxygen isotope equilibrium with the ambient seawater and not affected by the physiology of the animal [31]. Given that annual variations in salinity are very limited and seawater temperature has a broad annual range at SPM, δ^18^O variations in *S*. *solidissima* shells are expected to reflect the annual seawater temperature cycle. Thus, shell oxygen isotope-derived water temperature estimates were used to reconstruct the growth dynamics of *S*. *solidissima*.

Two *S*. *solidissima* specimens, S-SPM4-06112015-2 and S-SPM4-06112015-16 (hereafter referred to as shell #2 and #16, respectively), with a maximum shell length of 130 mm and 140 mm, respectively, were analyzed between their third and fourth year of growth in calendar years 2010 and 2011. These two specimens were selected because they were the youngest in our shell collection, with the exact same age. Shell aragonite samples were collected from thick cross-sections using an automated high-resolution micro-sampling device (MicroMill, New Wave Research) equipped with a 300-μm conical drill bit (model H71.104.003, Gebr. Brasseler GmbH & Co. KG). Between 32 and 47 samples were drilled from each annual growth increment with an average distance between successive samples of 500 μm. A total of 165 discrete aragonite samples weighing 59–100 μg were collected. All samples were analyzed on a Thermo Finnigan MAT 253 continuous flow—isotope ratio mass spectrometer coupled to a GasBench II at the Institute of Geosciences of the University of Mainz (Germany). Stable oxygen isotope ratios were reported relative to the Vienna Pee-Dee Belemnite (VPDB) standard based on a NBS-19 calibrated IVA Carrara marble (δ^18^O = –1.91‰). The internal precision, based on eight injections per sample, was 0.04‰. The long-term accuracy (external precision) of the mass spectrometer based on 421 NBS-19 measurements over 1.5 years was better than 0.04‰. For reasons described by Füllenbach et al. [32], the shell δ^18^O values were not corrected for the different acid fractionation factors of the samples (aragonite) and standards (calcite).

In order to relate δ^18^O to past temperatures, we used a fractionation equation written by Grossman and Ku [33] and calibrated for biogenic aragonite, to which we applied the small modification required for δ^18^O_seawater_ described by Sharp [34]:

$$T({}^{\circ}C)=20.6-4.34\times \left(\delta^{18}O_{aragonite}-(\delta^{18}O_{seawater}-0.27)\right)$$

The temperature range covered by this equation (2.6–22.0°C) is consistent with the seawater temperature measured at SPM during the main growing season for *S*. *solidissima*. This paleo-temperature equation was also used by Ivany et al. [21] in the last study addressing *S*. *solidissima* δ^18^O_shell_ composition. As δ^18^O_seawater_ has not yet been measured at the sampling site, we used an average and constant δ^18^O_seawater_ value of -1.66‰ VSMOW calculated using an equation determined by LeGrande and Schmidt [35] for the North Atlantic and an annual average salinity of 31.49 ± 0.03 measured at a water depth of 0–5 m during the growing season.

We calculated error propagation in our estimation of seawater temperature (reconstructed from d18Oshell variations), considering (1) uncertainty (1σ) of the mass spec measurements, (2) 1σ of the slope of the d18Oseawater/S equation, (3) 1σ of the intercept of the d18Oseawater/S equation, and (4) 1σ of the salinity variations in the area. The resulting uncertainty is ±0.75°C.

### Sclerochronological analysis

The main objective of sclerochronological analysis was to compare the growth rates of the three anatomical parts. Similar to other bivalves, the growth rate of *S*. *solidissima* varies from year to year and exponentially decreases throughout ontogeny. This ontogenetic trend can be mathematically estimated by a growth equation. In the present study, the generalized von Bertalanffy growth function (gVBGF) was chosen because of its biological meaning [36]. The growth model was fitted to size-at-age data for each anatomical part of the 27 individuals using the following equation:

$$L{\left(p\right)}_{t}=L{\left(p\right)}_{\infty }*{\left(1-e^{-K(t-t_{0})}\right)}^{D}$$

Where *L(p)*_*t*_ is the predicted shell length (in mm) at time *t* (in years), *L(p)*_*∞*_ is the length reached after an infinite time of growth (in mm), *K* is the Brody growth constant defining the "speed" of growth (per year), *t*_*0*_ is the theoretical age at which the size would be zero (in years), and *D* determines the shape of the curve (more or less sigmoid). In order to remove this ontogenetic trend, growth indices (GIs) were calculated for each year and each anatomical part of each individual by dividing the measured increment width by the predicted increment width [31] as follows:

$$GI_{t}=\frac{L_{t+1}-L_{t}}{L{\left(p\right)}_{t+1}-L{\left(p\right)}_{t}}$$

Where *GI*_*t*_ is the growth index at *t* (in years), *L*_*t+1*_ *–L*_*t*_ is the measured shell increment at *t*, and *L(p)*_*t+1*_ *–L(p)*_*t*_ is the predicted shell increment length at the same time *t*. Individual time-series of GI were then standardized as follows [31]:

$$SGI_{t}=\frac{GI_{t}-\mu }{\sigma }$$

Where μ is the average of all GI values and *σ* the standard deviation. The standardized growth index (SGI) is a dimensionless measure of how growth deviates from the predicted trend. Positive values represent greater than expected growth, whereas negative values represent less than expected growth. Finally, the mean SGI and standard error were calculated for each year and each anatomical part to create three SGI chronologies.

### Statistical analysis

The robustness of the three SGI chronologies was tested. A frequently used assessment of the robustness of composite chronologies is the expressed population signal (EPS) [37], which is expressed as:

$$EPS=\frac{n*R_{bar}}{\left(n*R_{bar}+(1-R_{bar})\right)}$$

Where *R*_*bar*_ is the average of all correlations between pairs of SGI chronologies and *n* is the number of specimens used to construct the stacked chronology. EPS > 0.85 indicates that the variance of a single SGI chronology sufficiently expresses the common variance of all SGI series.

To measure the ordinal association between two anatomical parts (chondrophore–external layer; external layer–ligament; ligament–chondrophore), the Kendal rank coefficient correlations of the SGI chronologies were calculated. All statistical analyses were performed using R statistical analysis software [38].

### Regional growth comparison

A specialized von Bertalanffy growth function (sVBGF) for *S*. *solidissima* at SPM was applied to 532 size-at-age data pairs of the external layer using the following equation:

$$L{\left(p\right)}_{t}=L{\left(p\right)}_{\infty }*(1-e^{-K(t-t_{0})})$$

As age *versus* size relationships are always given in the literature in terms of age at a given shell length using sVBGF, we converted our curvilinear shell height data to shell length data before applying the same growth model. This conversion was achieved using the average curvilinear height:length ratio (0.92) of all 27 surfclams used in this study [19].

A direct comparison of growth patterns calculated in previous studies using the two parameters *L(p)*_*∞*_ and *K* may be mathematically feasible, but is not biologically consistent, as *K* negatively correlates with *L(p)*_*∞*_. Pauly [39] was the first to develop the concept of overall growth performance (OGP) to make individual growth comparable. Pauly and Munro [40] later introduced a closely related index of OGP (Φ') that is derived from the sVBGF as follows:

$$\phi \prime =\log K+2\;\log\left(0.1*{L(p)}_{\infty }\right)$$

Where *L(p)*_*∞*_ is in mm and *K* given per year.

Finally, we compared the growth parameters and OGP index of the SPM population to those of other populations along a latitudinal gradient from the Gulf of St. Lawrence in Canada (Prince Edward Island [41]—Northumberland Straight [42]—Magdalen Islands [43, 44] to the Northeast coast of the USA (Southern New England, New Jersey, Delmarva [45, 46]).

## Results

### Environmental monitoring

**Fig 3:**
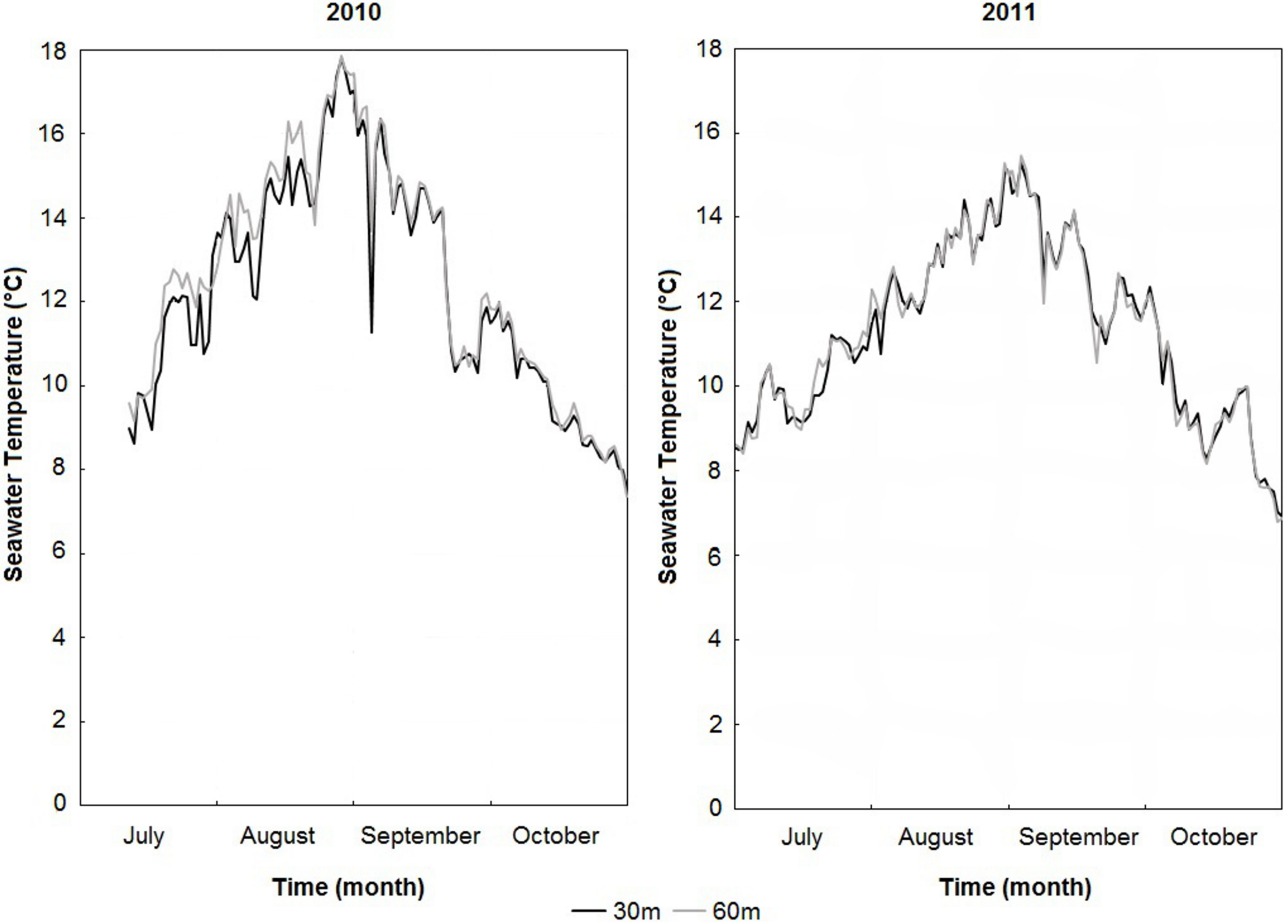
Seawater temperature variation at Miquelon Bay outlet. Measurements were made at a depth of 5 m on ropes moored at 30 m (grey curves) and 60 m (black curves). Values are presented as daily means during two periods from 10/07/2010 to 31/10/2010, and from 01/07/2011 to 31/10/2011.

Seawater temperatures recorded in 2010 and 2011 revealed a strong seasonal pattern, ranging from 0.95°C (28/03/2011) to 17.85°C (29/08/2010). We clearly saw two different thermal profiles in 2010 and 2011 (Fig 3). Higher temperatures were observed in 2010, especially during the temperature increase phase. Between mid-July (15/07) and early September (04/09), we observed an average daily difference of 1.7°C between the two years. This difference was less obvious during the second part of the year (05/09 until 04/11), with the average daily temperature of 2010 being only 0.4°C warmer than during 2011.

**Fig 4:**
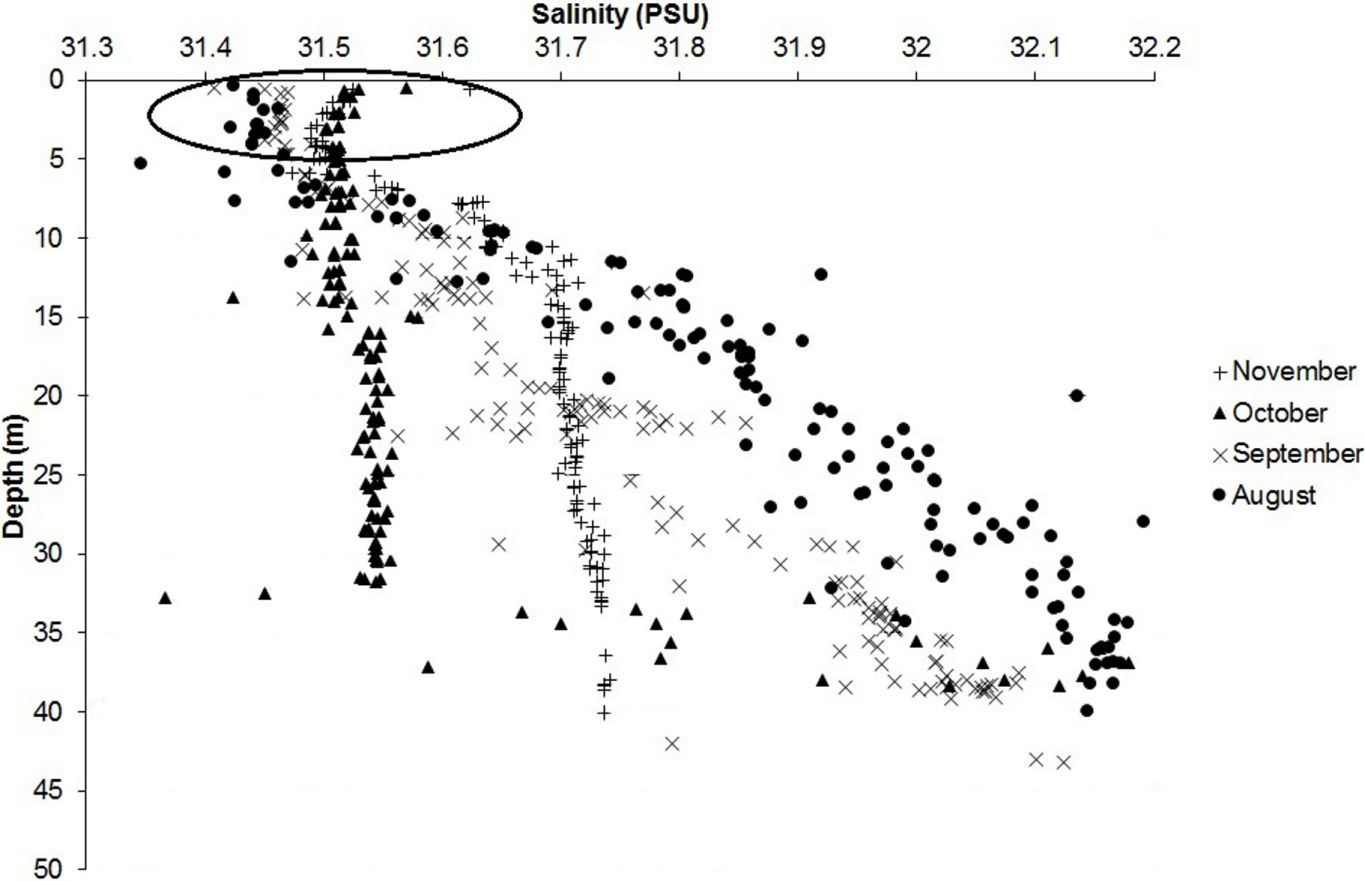
Salinity measured along a vertical profile at a water depth of 0–40 m on 04/08/2010, 15/09/2010, 04/10/2010, and 16/11/2010. The circled points are those between 0 and 5m which were used to calculate the mean salinity used to reconstruct the δ^18^O_seawater_.

Mean salinity at a depth of 0–5 m was calculated from 85 measurements recorded between the beginning of August 2010 and the middle of November 2010 near the *S*. *solidissima* collection site and found to be 31.49 ± 0.03 (Fig 4).

### Oxygen isotope composition of shells

**Fig 5:**
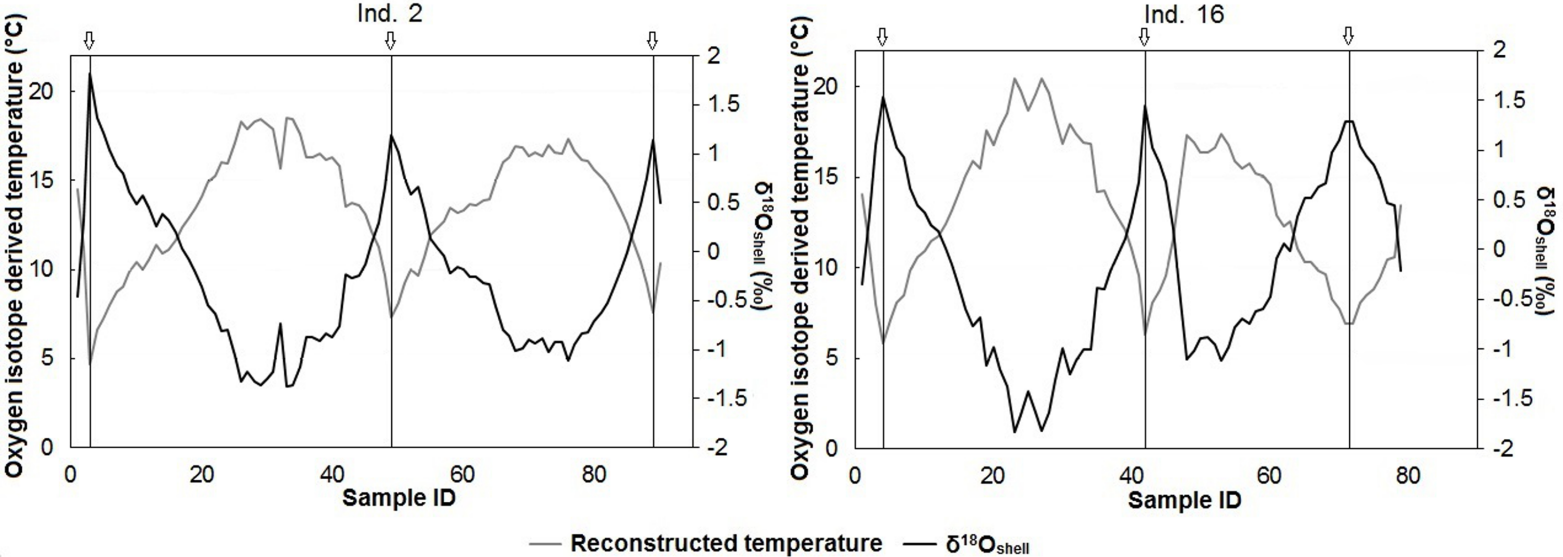
Comparison of δ^18^O_shell_ (black) and the reconstructed temperature (grey) for two *S*. *solidissima* shells. Shells #2 and #16 were sampled from the end of the second year of life toward the beginning of the fifth year. Vertical lines placed under the arrows indicate the position of shell growth lines.

The oxygen isotope profiles obtained from the third and fourth year of growth (2010 and 2011) of shells #2 and #16 were characterized by distinct seasonal variations in δ^18^O_shell_. Maximum δ^18^O_shell_ values occurred at the exact same position in both specimens, i.e., at major growth lines (Fig 5), confirming that they are formed annually and that shell growth ceases during the winter. The δ^18^O_shell_ values fluctuated around a mean of ≈ 0‰. The largest δ^18^O_shell_ amplitudes of 3.35‰ and 3.18‰ were observed in 2010. This amplitude decreased considerably in 2011 to a low of 2.40‰ and 2.30‰ (Fig 5).

Seawater temperatures calculated from the δ^18^O_shell_ values were between 4.7°C and 20.5°C. These temperatures agree with instrumentally recorded temperatures. Moreover, the observed cyclicality is consistent with the position of the annual growth lines (Fig 5). In all studied years for both specimens, annual growth lines were formed when the temperature was very close to the seasonal minimum. However, there were some differences between the two specimens. Despite similar shapes of the reconstructed temperature curves, in 2010, shell #16 recorded higher temperatures (20.5°C) than shell #2 (18.5°C). This offset falls within the uncertainty of temperature reconstruction and is therefore likely not significant. As sampling spots for isotope analysis were evenly spaced, the number of δ^18^O_shell_ measurements during the phases of temperature increase and decrease provides a semi-quantitative estimate of the seasonal shell growth rate. In three of four cycles, the growth rate was higher during the summer than during the fall (Fig 5), with the only exception occurring in 2011 (shell #16).

### Sclerochronological analysis

All subsequent results were calculated under the assumption of annual growth line formation, as confirmed by δ^18^O analysis. Growth analyses were performed on 27 individuals with ontogenetic ages of 8 to 27 based on annual increment counts in the three anatomical parts: the outer shell layer, the resilium, and the chondrophore.

**Fig 6:**
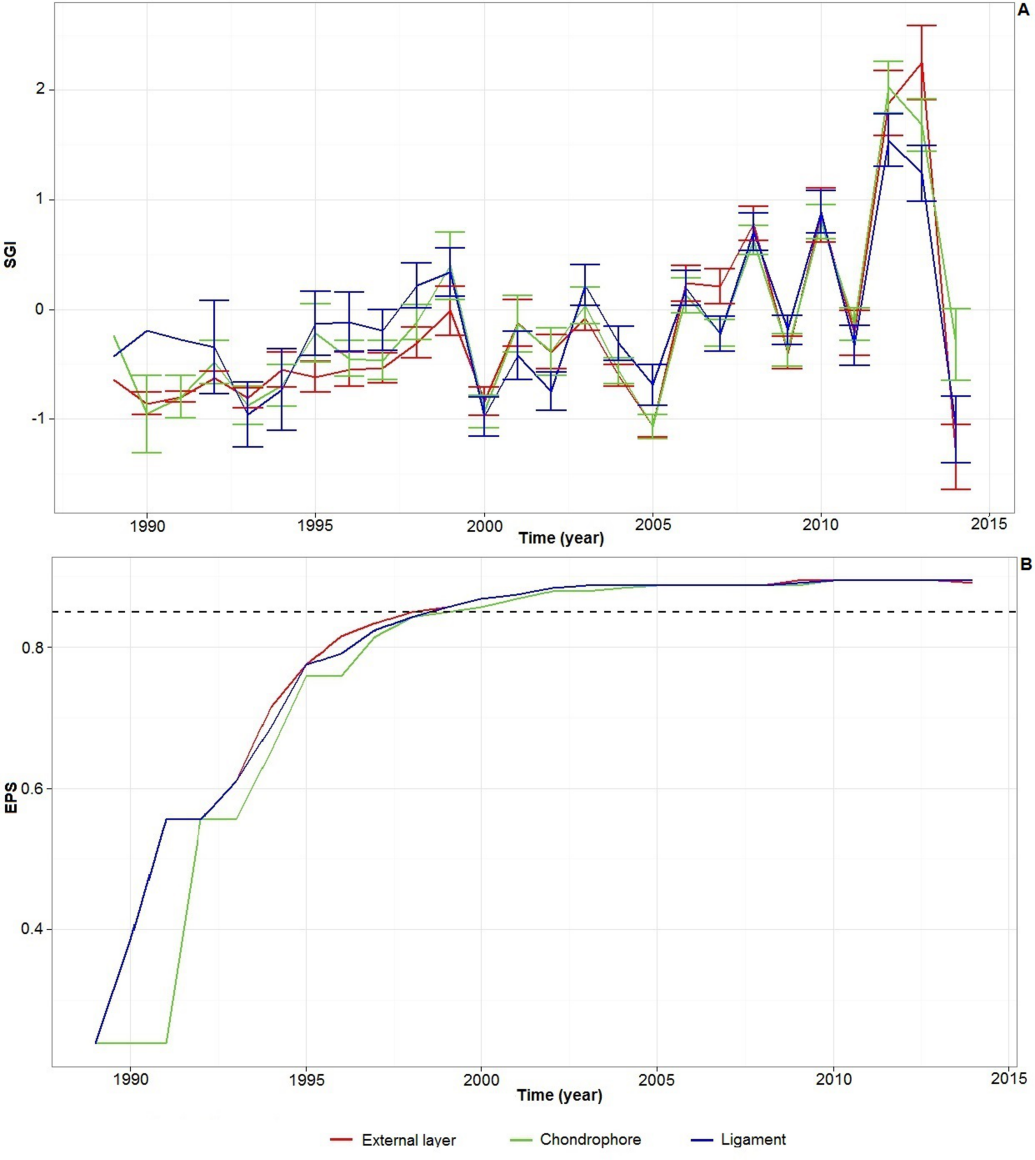
SGIs and EPS values. **A**) Mean annual SGI and associated standard errors calculated from 27 live-collected *S*. *solidissima* during the period 1989–2014. **B**) Expressed population signal (EPS) values associated with each anatomical part SGI series.

The mean annual SGI values and standard errors are shown in Fig 6. Before 1998, the EPS values of all anatomical parts remained below the critical threshold of 0.85. Therefore, the stacked SGI chronologies were robust only during the period 1998–2014 (sample depth > 16 shells).

Pairwise correlations calculated on SGI time-series were significant (*p* < 0.001) between the external layer and chondrophore (*τ* = 0.68), between the ligament and chondrophore (*τ* = 0.65), and between the external layer and ligament (*τ* = 0.55).The three SGI curves follow the same zigzag pattern, with an alternation of high and low values approximately every second year, which is very clear after the calendar year 2000 (Fig 6). The mean annual SGI varied considerably over the 25-year period, ranging from a minimum of -1.37 in 2014 to a maximum of 2.36 in 2013. The highest mean annual SGI variations were observed for the external shell layer. Notably, inter-annual SGI variations increased after 2005 and were at a maximum between 2013 and 2014 (Fig 6).

### Regional growth comparisons

**Fig 7:**
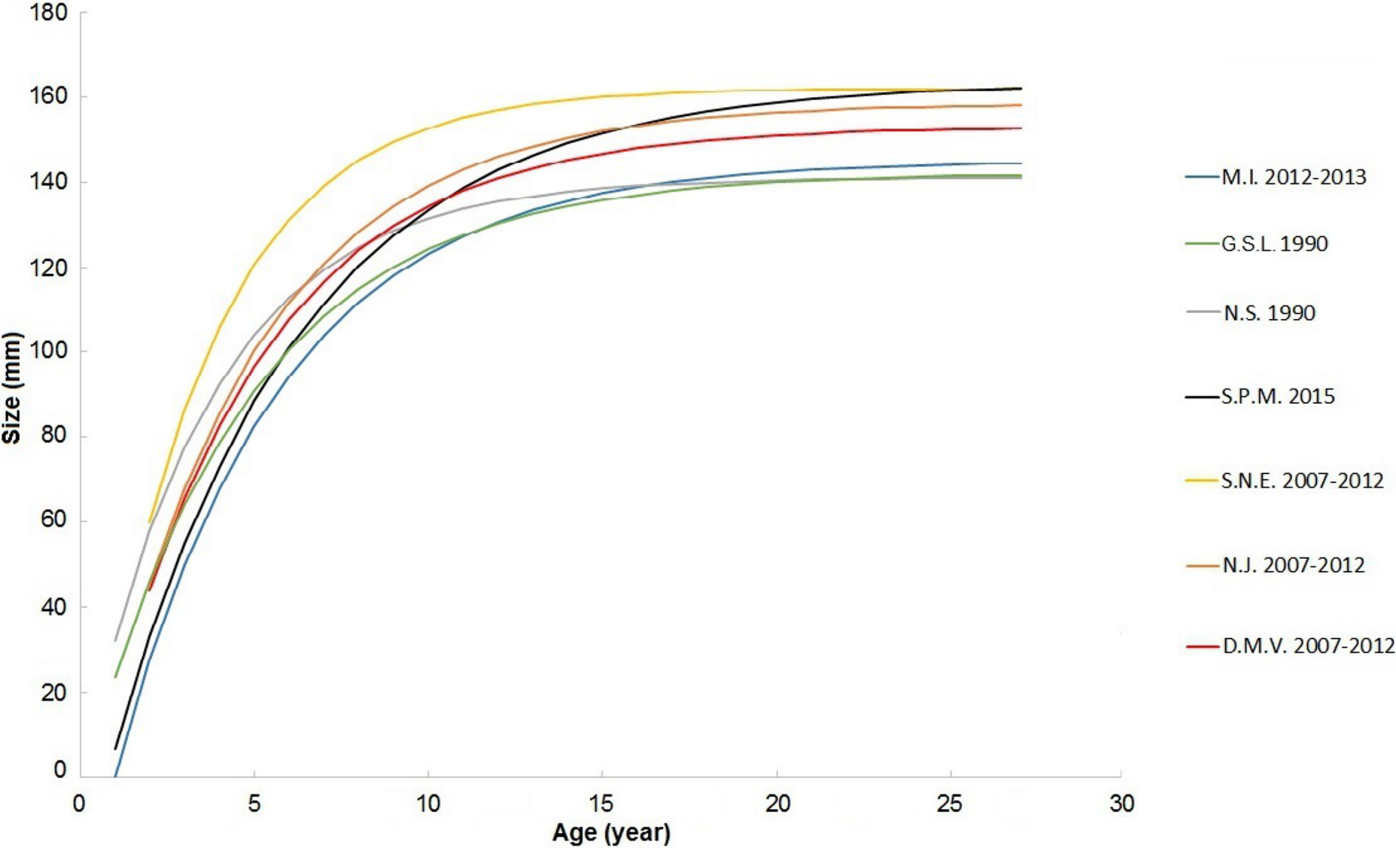
sVBGF curves in the last study from each geographic zone (details in Table 1).

**Fig 8:**
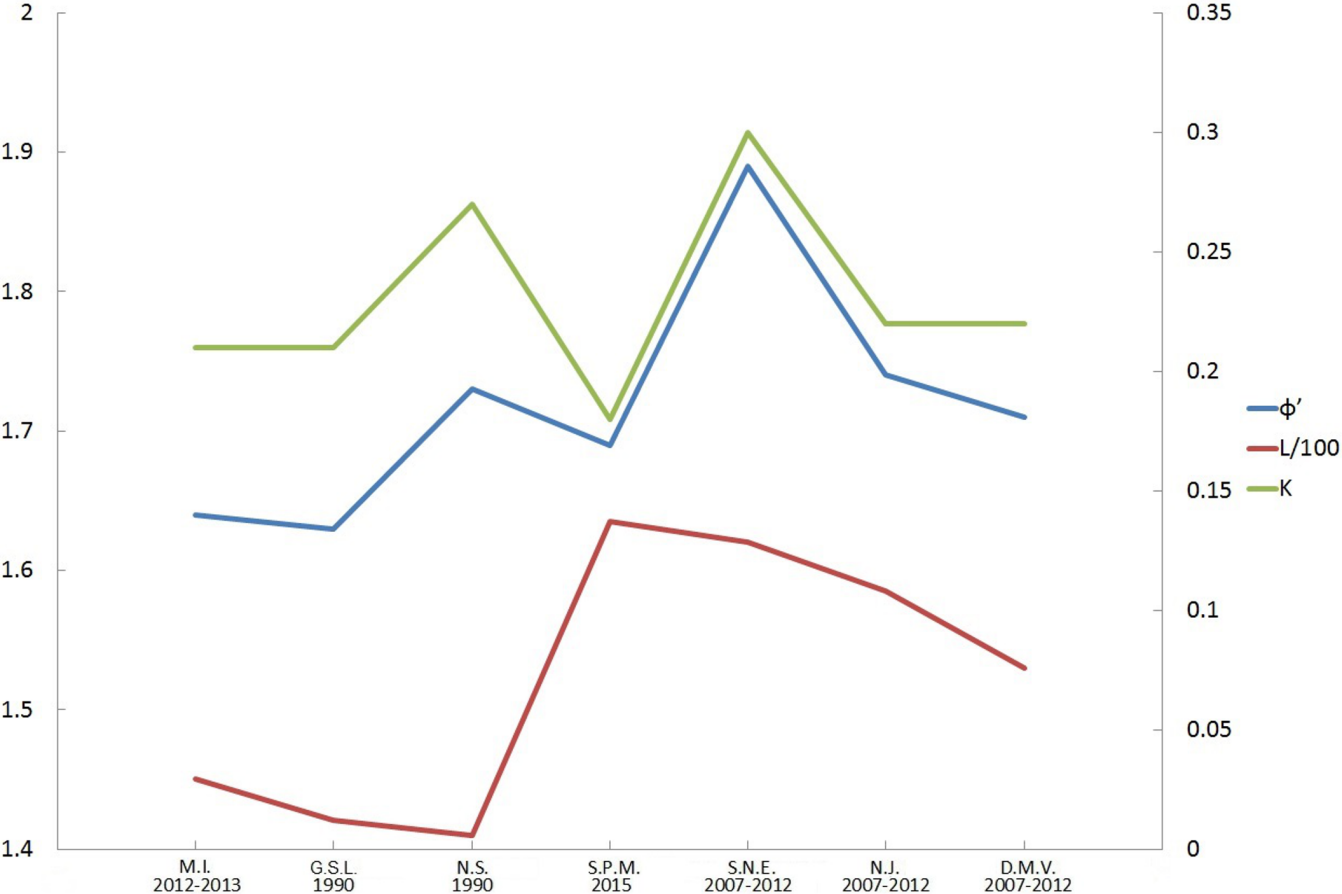
*L*_*∞*_/100, *K* and overall growth performance (*ɸ’*) calculated using the sVBGF parameters of the last study from each geographic zone (details in Table 1).

According to the specialized von Bertalanffy growth function applied to growth data from the SPM population, *L*_*(p)∞*_ was 163.5 mm, *K* 0.18, and *t*_*0*_ 0.77 (Table 1). Even if this growth function fits this size-at-age dataset almost perfectly (*R*^*2*^ = 0.96), important variations in growth rates were observed between individuals. The growth dynamics of the SPM *S*. *solidissima* population are particularly close to those of Northumberland Straight [42], making it closer to Canadian populations. A distinct difference exists between US and Canadian surfclam populations in terms of growth dynamics (Fig 7). The growth constant *K* is higher in the US than in Canada or at SPM except for GSL 1990 (Fig 8). Thus, American surfclams reach the asymptotic phase of shell growth faster than the Canadian or SPM populations. Regarding the *L*_*∞*_, the only population that differs from the others is from Magdalen Islands, with a *L*_*∞*_ value 2 or 3 cm lower than the others (Table 1).

**Table 1:** Specialized von Bertalanffy growth parameters (*L*_*∞*_, *K*) and overall growth performance index (*ɸ’*) from various studies on *S*. *solidissima* shell growth along the northeastern American coast, from the Gulf of St. Lawrence (GSL, Canada) to Delmarva (USA).

Site	Year	*L*_*∞*_	*K*	*ɸ’*	Method	Reference
GSL (Magdalen Islands)	1986	133	0.2	1.55		Gendron 1988 [43]
GSL (Magdalen Islands)	2012–2013	145	0.21	1.64	Chondrophore and/or outer shell layer	Brulotte 2016 [44]
GSL (N-Prince Edward Island)	1984	142.1	0.21	1.63	Chondrophore	Sephton and Bryan 1990 [41]
GSL (Northumberland Straight)	1981	172.2	0.15	1.65	Shell growth ring measurement following Kerswill 1944 [18]	Roberts 1981 [42]
GSL (Northumberland Straight)	1984	141	0.27	1.73	Chondrophore	Sephton and Bryan 1990 [41]
Saint-Pierre and Miquelon	2015	163.5	0.18	1.69	Outer shell layer	Present study
Southern New England	1980	166.5	0.3	1.93	Chondrophore	Weinberg and Helser 1996 [45]
Southern New England	1989–1992	165.4	0.31	1.90	Chondrophore	Weinberg and Helser 1996 [45]
Southern New England	2007–2012	162	0.3	1.89	Chondrophore	Chute et al. 2016 [46]
New Jersey	1980	170.8	0.25	1.87	Chondrophore	Weinberg and Helser 1996 [45]
New Jersey	1989–1992	163.7	0.22	1.76	Chondrophore	Weinberg and Helser 1996 [45]
New Jersey	2007–2012	158.5	0.22	1.74	Chondrophore	Chute et al. 2016 [46]
Delmarva	1980	171	0.26	1.87	Chondrophore	Weinberg and Helser 1996 [45]
Delmarva	1989–1992	164	0.18	1.68	Chondrophore	Weinberg and Helser 1996 [45]
Delmarva	2007–2012	153	0.22	1.71	Chondrophore	Chute et al. 2016 [46]

The OGP index (*ɸ’*) was lower for the three Canadian populations (~1.65), slightly increased at SPM, and reached a maximum value of 1.9 on the coast of southern New England. *ɸ’* decreased to approximately 1.7 close to the southern limit of the distribution area of *S*. *solidissima* (Fig 8).

## Discussion

### Isotopic validation of annual banding

The two isotopic profiles obtained from the third and fourth years of growth (2010–2011) of two *S*. *solidissima* shells from SPM agreed well with each other. The correspondence between the dark growth lines and δ^18^O_shell_ maxima confirms the findings in previous studies [9, 19] reporting that *S*. *solidissima* forms annual shell growth increments during the warmest months. Sea surface temperature measurements (top 5 m of the water column) showed that 2010 was a bit warmer than 2011, which was reflected by a higher δ^18^O_shell_ amplitude in the shells of both specimens. In the absence of δ^18^O_seawater_ data from the sampling site, we used a constant δ^18^O_seawater_ value of -1.66‰. This value was calculated using the equation from LeGrande and Schmidt [35] for the North Atlantic basin and an average salinity of 31.49, which was measured at SPM in the upper 5 m of the water column between August and November 2010. Reconstructed seawater temperatures were 4.7°C to 20.5°C. According to these results, there is an offset of a few degrees between measured and reconstructed temperature maxima. This offset can be explained by a difference in the thermal profiles between the localities at which the measurements were made, or by a difference in the δ^18^O_seawater_ value. Combined with the instrumental measurements of seawater temperature, these results allow us to determine the growing season of *S*. *solidissima* during its third and fourth years of growth at SPM. Because the reconstructed temperature minimum occurred only once in the chronologies (in shell #2: 4.7°C during winter 2009/2010) and >90% of shell growth occurred when the seawater temperature was >8°C, we can assume that the main growing season during the third and fourth year of life occurred between the end of June and the end of October. For comparison, Ivany et al. [21] showed that, during the third year of growth, *S*. *solidissima* from New Jersey grew for 72.3% of the year, i.e., the growing season was almost twice as long as at SPM (for a comparable annual growth of slightly over 20 mm in both cases).

Similar observations have been made for pectinid bivalves of various species living in contrasting environments. For example, Heilmayer et al. [47] accumulated strong empirical evidence that a lower metabolic rate, a measure of the energy consumed by vital functions, including the maintenance and production of gametes, in colder environments reduces the energy cost of maintenance. Thus, a larger fraction of metabolic energy can be allocated to growth-enhancing levels of growth performance and efficiency at lower temperatures. Moreover, except for shell #16 in 2011, oxygen isotope analyses revealed that shell growth at SPM occurs most rapidly during the first half of the year, between the beginning of July and the end of August. This finding is consistent with those of Jones et al. [9] and Ivany et al. [21], who also used stable oxygen isotopes and noted that growth of *S*. *solidissima* in New Jersey occurs most rapidly in spring and early summer, slowly in late summer, and extremely slowly or is non-existent in winter. The slowly decreasing δ^18^O_shell_ values for the earliest part of each increment could reflect the spring phytoplankton bloom when the increase in temperature is still small. The slowing of shell growth observed during fall could be associated with a metabolic strategy of *S*. *solidissima*; the energy assimilated by an organism during fall could be preferentially allocated to energy reserves for winter rather than shell production. To confirm this hypothesis, field studies are needed on phytoplankton dynamics and the physiology of *S*. *solidissima*. The growth anomaly observed during the phase of temperature increase in 2011 for shell #16 could be explained by an individual event, such as predation or disease. This is consistent with the presence of a disturbance line within this annual increment. Moreover, at the collection site we clearly observed the presence of many *S*. *solidissima* predators [13], such as naticid snails (*Euspira heros*), sea stars (*Asterias* spp.), and crabs (*Cancer irroratus*).

### Sclerochronological analysis

Analysis of the oxygen isotope composition of the outer shell layer of *S*. *solidissima* was useful to obtain insights into the seasonal timing of the growth line and increment formation. We also noted the occasional presence of disturbance lines in the outer shell layer (e.g., in shell #16 during summer 2011). The growth increment analysis in thin sections was also challenging due to erosion of the umbonal shell regions and constraints related to sample size and preparation techniques. This led us to investigate whether dark lines observed on the chondrophore and ligament cross-sections can provide an alternative record of the life-history traits of *S*. *solidissima*. Growth lines were used to calculate mean annual SGI values for each anatomical part studied. All pairwise correlations performed on these three SGI series were significant, suggesting that growth lines form annually in the external layer, chondrophore, and ligament of *S*. *solidissima*. Although the interest of using thin chondrophore sections for age and growth rate studies was highlighted previously for this species [48], we noted here that this hard part also exhibits disturbance lines. Growth lines in the resilium seem to be less ambiguous to read because of higher contrast (Fig 1). Moreover, identifying and counting growth lines in the ligament is straightforward and allowed us to save time, as no additional grinding or polishing was required. Another advantage of using ligament cross-sections is the composition of this archive, i.e., aragonitic crystals embedded in an organic matrix, making it less prone to breakage than other shell parts composed mainly of calcium carbonate. Although micrometer-scale chemical studies of the hinge ligament have not yet been conducted, we can assume that the composition of this archive could lead to the development of new proxies.

SGI time-series of all specimens and anatomical parts of *S*. *solidissima* with overlapping lifespans exhibited a high degree of running similarity, i.e., relative changes in annual shell growth were similar among different specimens and anatomical parts. The standardized growth record of *S*. *solidissima* at SPM shows that growth was greater than expected during the years 2008, 2010, 2012, and 2013, whereas growth was less than expected in 2000, 2005, and 2014.

In the present study, we found that 2010 was warmer than 2011. This is especially true during the first half of the growing season, which seems to be the most important period of growth for *S*. *solidissima* at SPM and other localities [9, 21]. This observation suggests that higher temperatures during summer are favorable for the growth of *S*. *solidissima* at SPM, which is expected for a species at the northern limit of its distribution area. However, during 2014, which was not reported to have an unusually cold summer, the shell grew slower than expected, suggesting that the annual shell growth of *S*. *solidissima* is governed by a combination of different external drivers and not only by seawater temperature. Additional environmental factors governing shell growth may include food quality and quantity [45, 7], temperature [49], salinity [49], and dissolved oxygen [45]. More detailed instrumental records of such environmental factors would be useful for better understanding the drivers of changed in shell growth at SPM.

### Regional growth comparisons

Growth is not uniform in the different settings in which *S*. *solidissima* is distributed. Such differences were observed previously along the east coast of the US [46]. Our study compared the shell growth of SPM populations with that of populations from the US and Canadian coasts to identify potential differences. The SPM population has some similarities with Canadian populations with regard to the parameters *K* and *ɸ’*. Slower growth (*K*) observed in two from three Canadian and SPM populations may be controlled, at least in part, by the environment. The environmental conditions are actually less favorable for growth in the northern and southern limits of the geographic range of *S*. *solidissima*, and previous studies have reported slower shell growth in southern populations [46]. Other possible sources of regional growth variation are genetic differences, as the only regional population with a distinctive genetic makeup comes from the Gulf of St. Lawrence [50]. All Canadian populations, differs from the others by low *L*_*∞*_ values. In terms of environmental conditions, SPM and the Canadian populations do not differ much. A major difference between these two sites is the historic surfclam fishery which occur in Canada, whereas the SPM archipelago is free of any *S*. *solidissima* exploitation. The low *L*_*∞*_ values observed for the Canadian surfclams may be at least partially induced by fishery activities because the largest specimens were preferentially removed from the population [51].

Other interesting features are the ontogenetic age and maximum shell length of inshore SPM surfclams used here. According to previous studies, *S*. *solidissima* tends to be smaller in size and to have a shorter lifespan in inshore populations. These trends have been attributed to high population density, differences in annual mean temperature, and more extreme temperature and salinity regimes [19, 52, 49]. However, based on the ages and sizes observed in this study, this does not seem to be the case at SPM, most likely due to its geographic position at the northern limit of the geographical distribution area of this species. This localization probably limits stresses related to high temperatures during summer. Moreover, the offshore position of SPM and the absence of major freshwater influx limits stress related to variations in salinity.

## Conclusions

This study demonstrates that surfclams from SPM have annual shell growth increments. In addition, *S*. *solidissima* shell growth at that site mostly occurs over a 4-month period between the end of June and the end of October. The three different methods used to investigate shell growth suggest that growth lines appear to form on a periodic annual basis in the outer shell layer, chondrophore, and ligament of *S*. *solidissima*. This suggests that ligament cross-sections can provide an accurate estimate of age and growth, which is less ambiguous to read and requires less preparation time than the chondrophore and outer shell layer. The SGI chronologies vary greatly between years, and these variations have been more important since 2005. However, these results are challenging to interpret because suitable data on the environment and physiology of this species are lacking. Finally, from a regional perspective, this population is interesting because of its geographic setting at the Gulf of Saint Lawrence outlet and due to the absence of commercial and recreational fisheries.
